# Supplementary material for: Structural insights into the mechanism of DNA branch migration during homologous recombination in bacteria
Source: EMBO J. 2024 Oct 18;43(23):6180–98. doi: 10.1038/s44318-024-00264-5 (PMC11612176; doi:10.1038/s44318-024-00264-5)
Supplement: Supplementary file 1 — Appendix [file 44318_2024_264_MOESM1_ESM.pdf]

Appendix for

Structural insights into the mechanism of DNA branch migration during homologous recombination in bacteria

Leonardo Talachia Rosa [1,3]#; Emeline Vernhes [2,4]#; Anne-Lise Soulet[2]; Patrice Polard [2]*; Rémi Fronzes [1]*

1. Structure and Function of Bacterial Nanomachines – Institut Européen de Chimie et Biologie, Microbiologie fondamentale et pathogénicité, UMR 5234, CNRS, University of Bordeaux, 2 rue Robert Escarpit, 33600, Pessac, France

2. Laboratoire de Microbiologie et de Génétique Moléculaire (UMR 5100). Centre de Biologie Intégrative; 169, avenue Marianne Grunberg-Manago; CNRS - Université Paul Sabatier - 31062 Toulouse cedex 09, France

3. Present address: Departamento de Bioquímica e Biologia Tecidual. Laboratório de Bioquímica de Complexos Bacterianos. Instituto de Biologia. Universidade Estadual de Campinas (UNICAMP)– Rua monteiro Lobato, 255 – Campinas-SP, Brasil 13083-862

4. Present address : TBI, Université de Toulouse, CNRS, INRAE, INSA, Toulouse, France

*correspondance : patrice.polard@univ-tlse3.fr, remi.fronzes@u-bordeaux.fr
# These authors contributed equally to this work.

CONTENT

Appendix Figures

Appendix Figure S1: CryoEM data collection, processing and modeling of RadA. 2
Appendix Figure S2: CryoEM data collection, processing and modeling of ComM hexamer bound to DNA. 3
Appendix Figure S 3 : Purified RadA and ComM used for cryo-EM and activity tests. 4
Appendix Figure S4: local refinement of DNA-bound ComM hexamer. 5
Appendix Figure S5: CryoEM data collection, processing and modeling of DNA-free ComM hexamer. 6
Appendix Figure S6: CryoEM data collection, processing and modeling of ComM dodecamer. 7
Appendix Figure S7: CryoEM processing workflow for RadA and ComM 8

Appendix Tables

Appendix Table S1: Synthetic oligonucleotides used for in vitro reconstitution of helicase hexamers ____ 9
Appendix Table S2: PDB-PISA analysis of RadA and ComM Lon domain hexamers ____ 9
Appendix Table S3: CryoEM data collection, processing and modeling statistics ____ 10

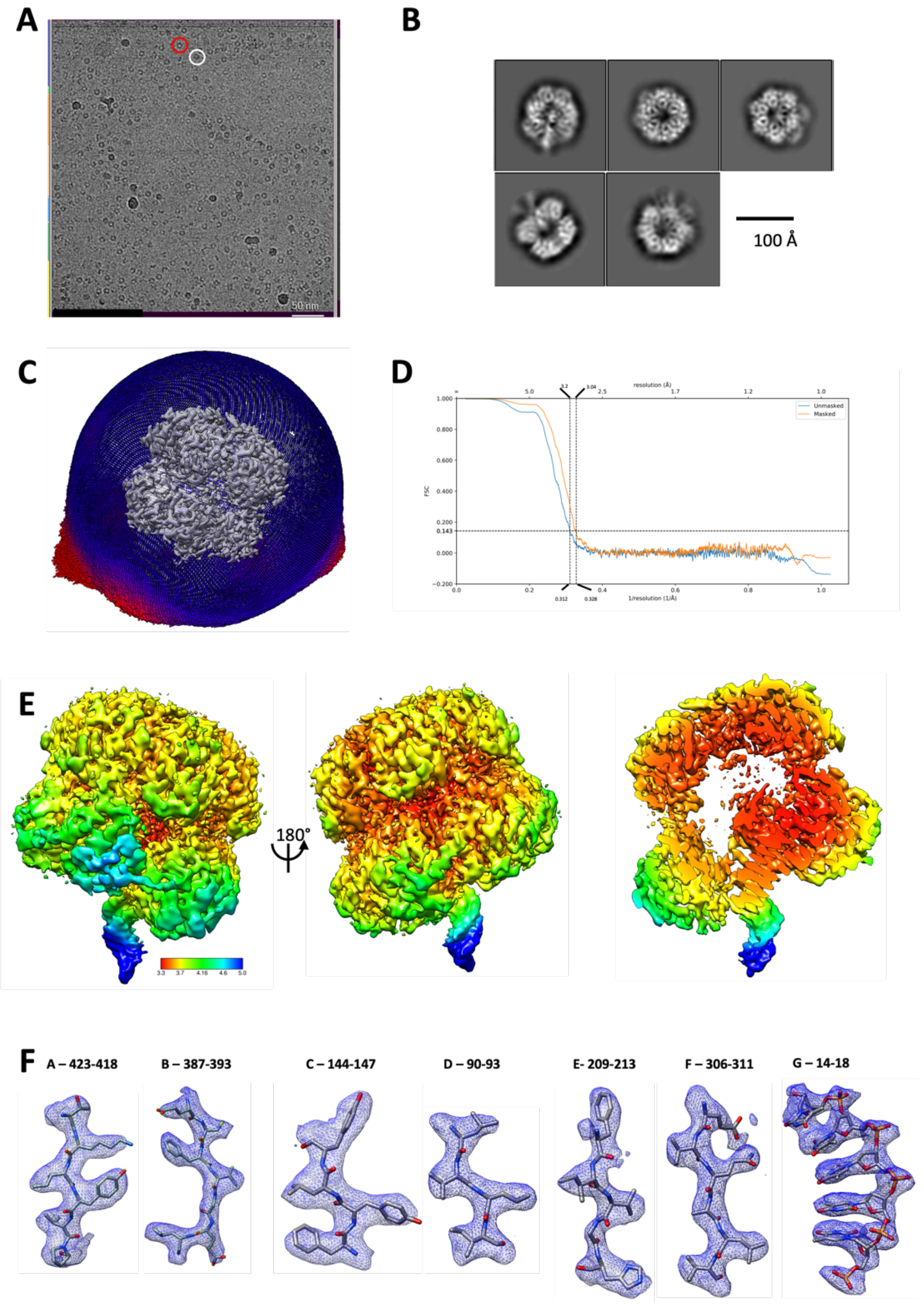

Appendix Figure S1: CryoEM data collection, processing and modeling of RadA.
A Representative micrograph for RadA dataset. Circle shows a typical top view of RadA, while the white circle represents a side view.
B representative 2D classes after particle classification in Relion 3.1
C Particle orientation distribution, plotted on the Euler sphere around a representation of the RadA CryoEM map
D Fourrier Shell Correlation (FSC) map obtained from half-maps in Phenix Autosharpen software.
E Local-filtered map of RadA at level 0.015, coloured by local resolution, with a slice of the central region of RadA (right panel).
F Representative regions of the CryoEM map depicted as a blue mesh, with respective model fit to the density. Chain identifier and residues number for the modelled regions are shown above each image.

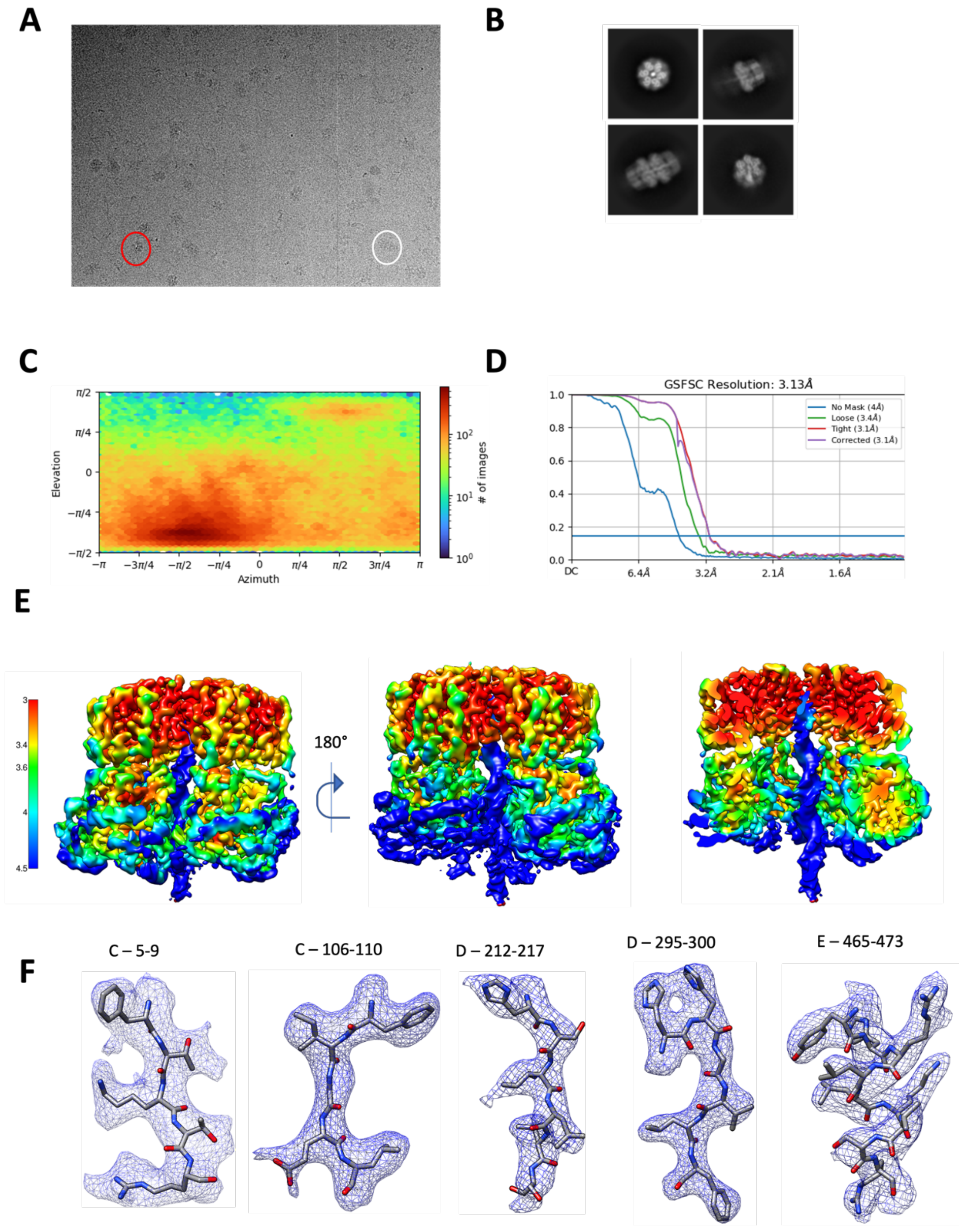

Appendix Figure S2: CryoEM data collection, processing and modeling of ComM hexamer bound to DNA.

A Representative micrograph for the collect on ComM. The red circle shows a typical top view of ComM, while the white circle represents a side view.

B Representative 2D classes after particle classification in Cryosparc.

C Particle orientation distribution of ComM focused map on DNA-bound ComM hexamer.

D Fourrier Shell Correlation (FSC) map obtained from half-maps in Cryosparc.

E Local-filtered map of ComM hexamer, at level 0.2, coloured by local resolution. Right panel shows a cross-section of the map.

F Representative regions of the CryoEM map depicted as a blue mesh, with respective model fit to the density. Chain identifier and residues number for the modelled regions are shown above each image.

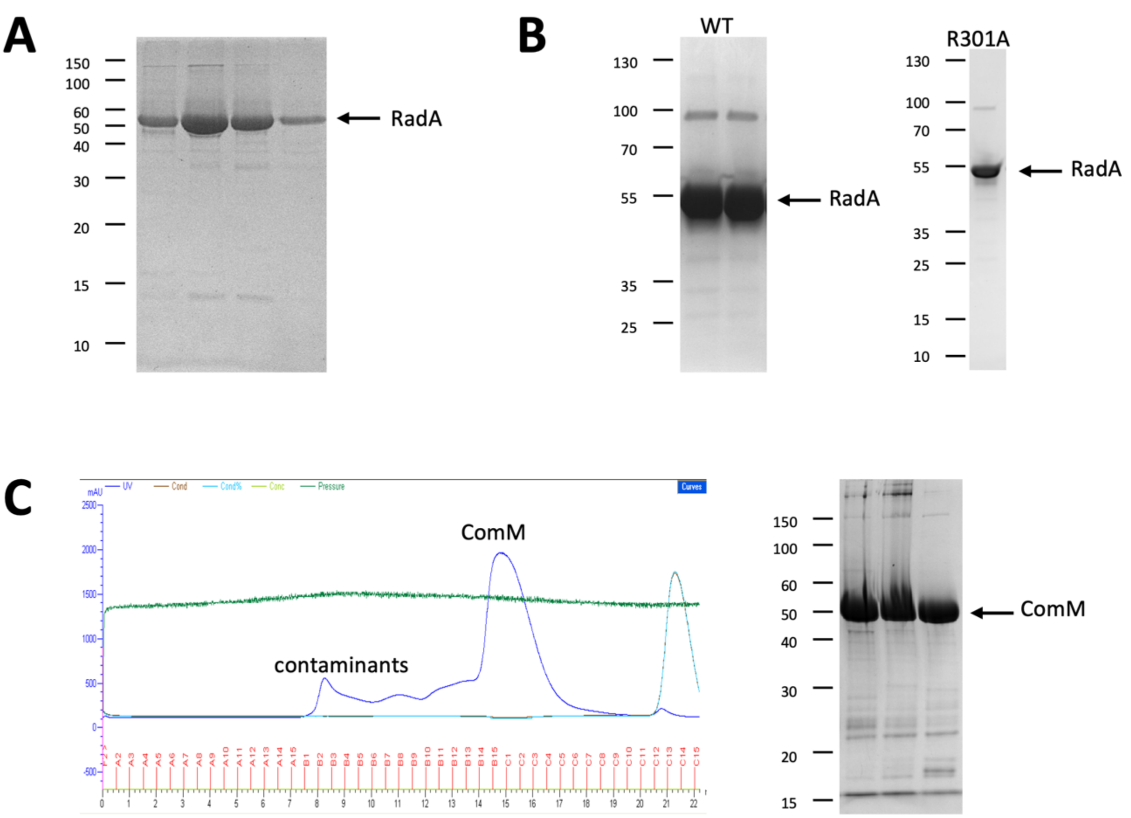

Appendix Figure S3 : Purified RadA and ComM used for cryo-EM and activity tests.

A SDS-PAGE of RadA gel filtration fractions used for cryoEM.

B SDS-PAGE of RadA chromatography fractions used for activity tests.

C ComM gel filtration chromatogram (left) and SDS-PAGE of fractions used for cryoEM (right).

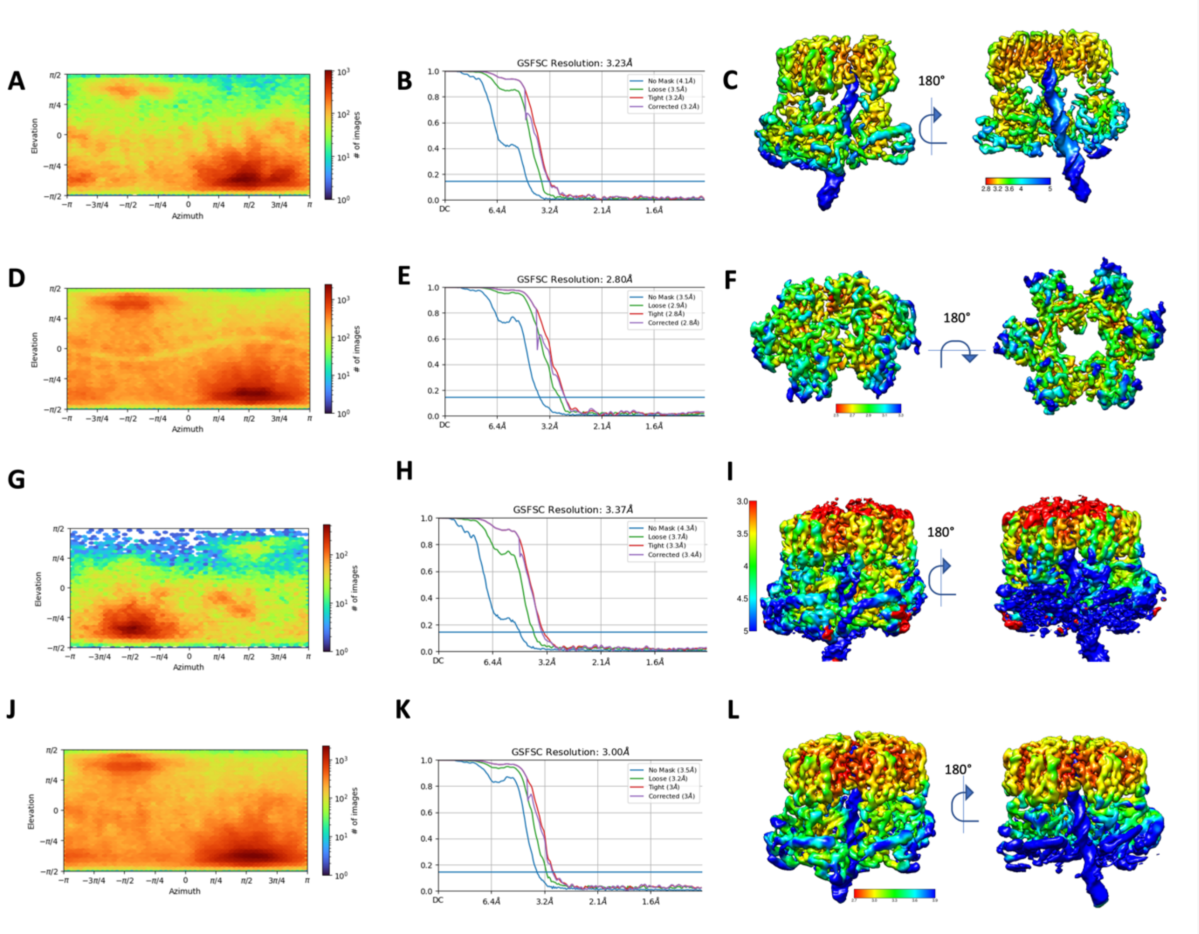

Appendix Figure S4: local refinement of DNA-bound ComM hexamer.

Local refinement on chains C-E + DNA (A to C).
A Particle orientation distribution of ComM focused map on chains C-E + DNA, obtained in Cryosparc
B Fourrier Shell Correlation (FSC) map obtained from half-maps in Cryosparc.
C Local-filtered map of ComM chains C-E + DNA, at level 0.07, coloured by local resolution.

Local refinement on the Lon domain (D to F).
D Particle orientation distribution of ComM focused map on Lon domains
E Fourrier Shell Correlation (FSC) map obtained from half-maps in Cryosparc.
F Local-filtered map of ComM Lon domains, at level 0.07, coloured by local resolution. Local refinement on the

Subpopulation 2 DNA-bound ComM hexamer (G to I).
G Particle orientation distribution of ComM focused map on Lon domains
H Fourrier Shell Correlation (FSC) map obtained from half-maps in Cryosparc.
I Local-filtered map of ComM Lon domains, at level 0.07, coloured by local resolution.

ComM consensus map used for subsequent focused refinement (J to L).
J Particle orientation distribution of ComM
K Fourrier Shell Correlation (FSC) map obtained from half-maps in Cryosparc for ComM consensus hexamer.
L Local-filtered map of ComM consensus map, at level 0.05, coloured by local resolution.

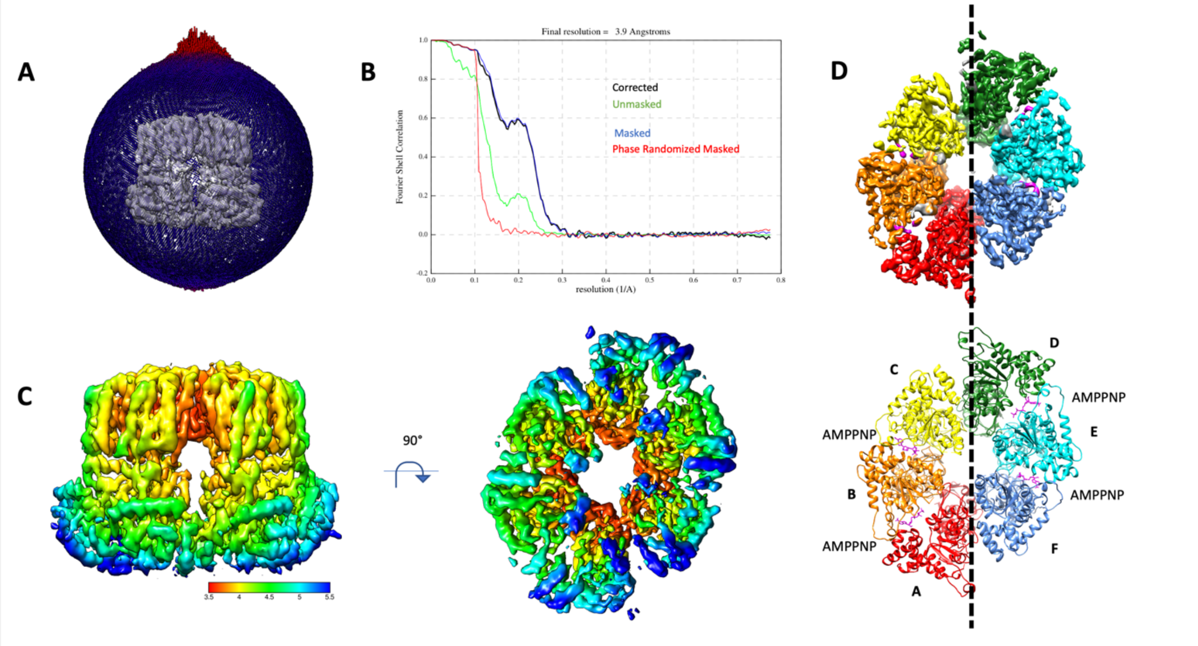

Appendix Figure S5: CryoEM data collection, processing and modeling of DNA-free ComM hexamer.

A Particle orientation distribution of DNA-free ComM hexamer, plotted in the Euler-angle sphere around the density map

B Fourrier Shell Correlation (FSC) map obtained from half-maps in Relion 4.0.

C Local-filtered map of DNA-free ComM hexamer, at level 0.002, coloured by local resolution.

D CryoEM analysis of DNA-free ComM hexamer. Despite unchanged position for the Lon domains (not shown), the ATPase domains are organized as a pair of trimers, showing C2 symmetry. Four molecules of AMP-PNP are orchestrated in the internal interfaces of each trimer (A-B, B-C, D-E, E-F), but not in the limit between the trimers (A-F and C-D). The 4 Å density map is depicted at level 0.002 (upper panel), coloured accordingly to the molecular model (lower panel).

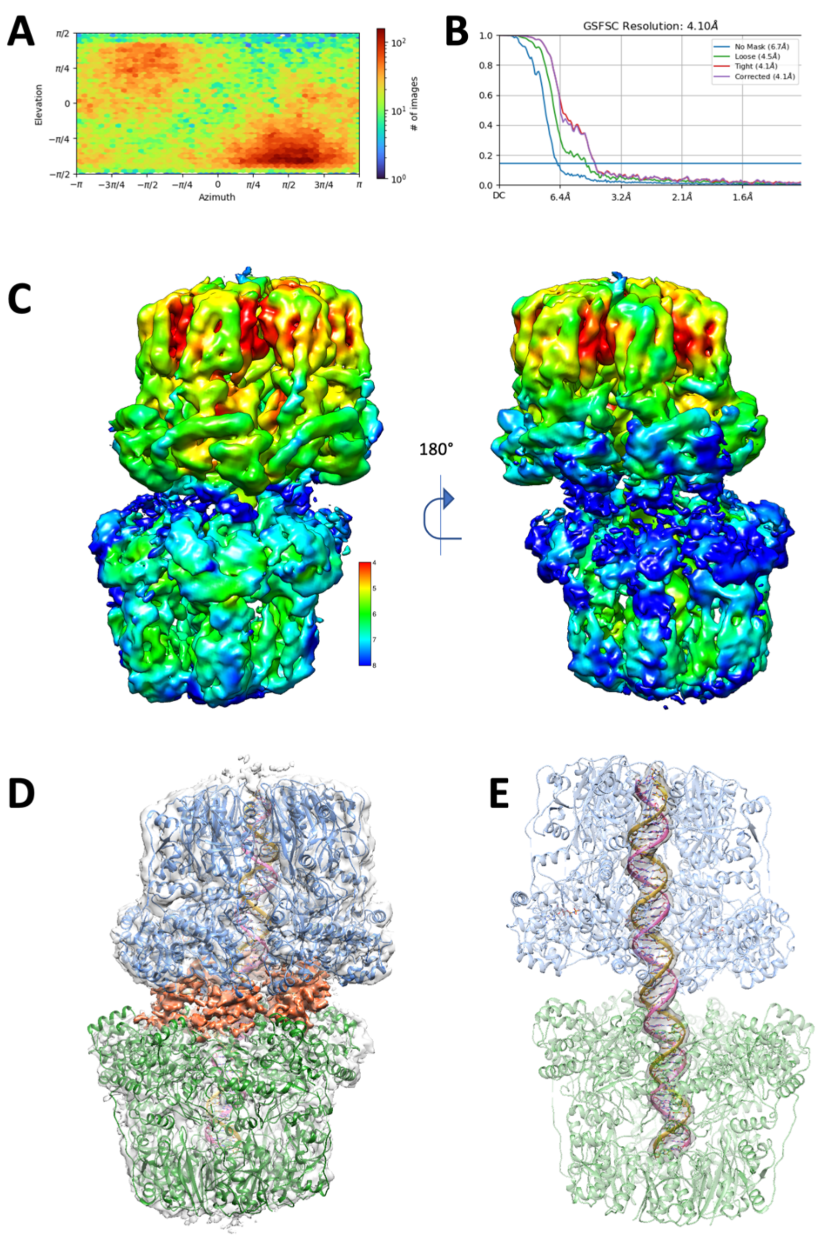

Appendix Figure S6: CryoEM data collection, processing and modeling of ComM dodecamer.

A Particle orientation distribution of ComM dodecamer

B Fourrier Shell Correlation (FSC) map obtained from half-maps in Cryosparc.

C Local-filtered map of ComM dodecamer, at level 0.04, coloured by local resolution.

D CryoEM density of ComM dodecamers, show with 70% transparency at level 0.04. Two ComM hexamers are fitted in the density, shown in blue and green ribbons, alongside a dsDNA molecule in magenta and golden encompassing the two hexamers. In between the fitted hexamers, an additional density is observed in the position equivalent to the expected C4 domain, highlighted in orange.

E Density corresponding to the 47 bp dsDNA encompassing both hexamers in the ComM dodecamer. Density for the DNA is shown at 50 % transparency while the rest of the density is hidden. Fitted dsDNA is depicted as ribbons in magenta and golden, with bases represented as sticks andcoloured by heteroatom. ComM hexamers are shown as blue and green ribbons, with 70% transparency.

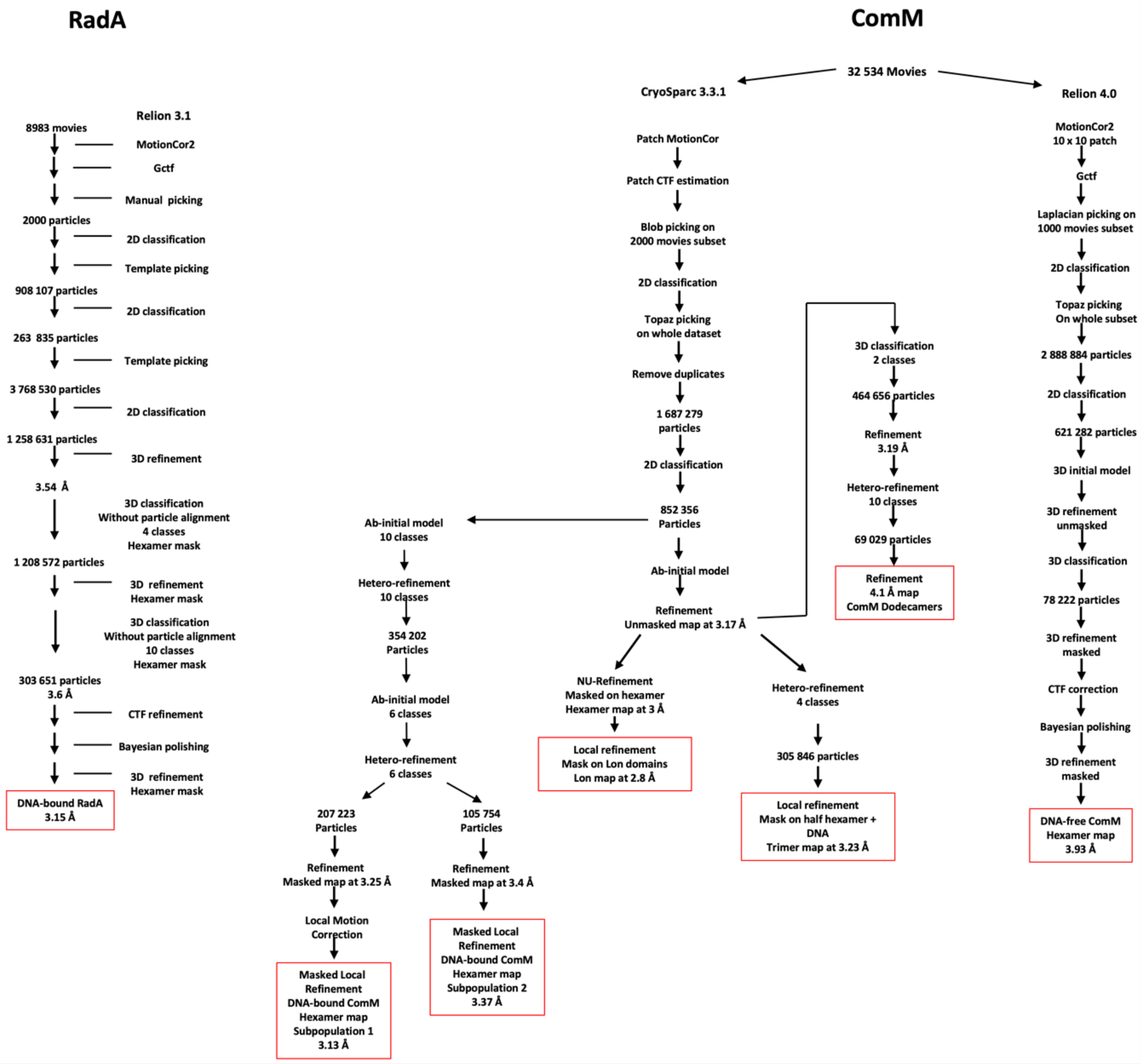

Appendix Figure S7: CryoEM processing workflow for RadA and ComM

Synthetic DNA sequences	
A	20A-60C
B	20T
C	30A-60C-30A
D	30-poly_dt
E	60A-60C
F	60A
G	60T
H	60G

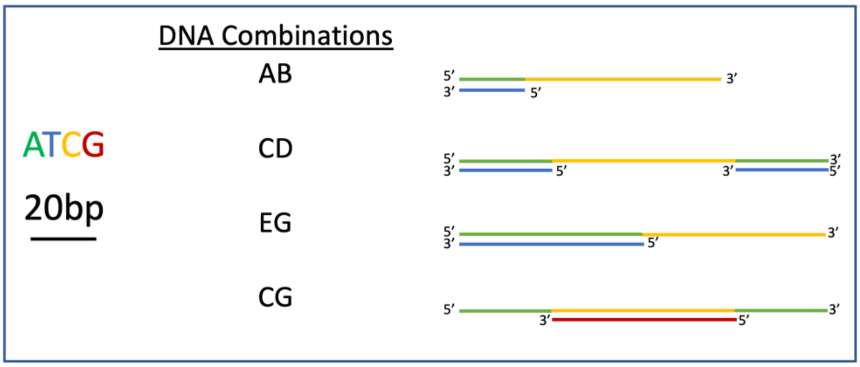

Appendix Table S1: Synthetic oligonucleotides used for in vitro reconstitution of helicase hexamers

	Solvent accessible surface	Number of interface residues	Interface area	Solvatation free energy	ΔG p-value*
				ΔG kCal/mol	
ComM	8206 Å2	21	678 Å2	-3.9	0.477
RadA	8947 Å2	32	1028 Å2	-6.8	0.28

Appendix Table S2: PDB-PISA analysis of RadA and ComM Lon domain hexamers

	DNA-bound ComM hexamer-Main Map	DNA-bound ComM hexamer-subpopulation 2	DNA-bound ComM hexamer for focused maps	ComM Trimer	ComM Lon domains	DNA-free ComM hexamer	ComM Dodecamer	DNA-bound RadA
PDB	7Z5V			7YXS	7YYD	7YXQ		7R5U
EMDB	14524	14523	14373	14362	14372	14360	14374	14339
**<u>Data collection</u>**								
Microscope	Titan Kryos							Titan Kryos
camera	Quantum K3							Falcon 3
Voltage (kV)	300							300
Magnification	130 000							165 k
Electron exposure (e– per Å2)	1.339							1.2
Total Dose	53.56							54.05
Pixel size (Å)	0.645							0.827
Decofus range (um)	-0.6 to -1.8							-0.5 to -2.5
**<u>Processing</u>**								
Symmetry imposed	C1							C1
Micrographs number	32 534							8983
Initial particle images (no.)			1 313 661			1 249 471	1 313 661	3 768 530
Final particle images (no.)	207 223	105 754	852 356	305 846	852 356	78 222	69 029	97 546
Map resolution (Å)–(0.143 FSC threshold model)	3.13	3.37	3	3.23	2.8	3.93	4.1	3.15
**Refinement and validation**								
Map sharpening (B-factor) (Å–2)	80.6	75.8	117.12	103.1	92	82	58	101
**<u>Model composition</u>**								
No. of chains	12			9	6	10		14
Atoms (no.)	23172			12162	16267	22258		19206
Aminoacid Residues (no.)	2898			1446	1080	2891		2376
Nucleotide Residues	48			48	0	0		42
Ligands (no.)	4			3	0	4		10
Bond lengths (Å)	0.005			0.003	0.003	0.004		0.004
Bond angles (°)	0.734			0.653	0.609	0.846		0.713
Ramachandran favored %	90.86			92.17	93.07	93.01		94.75
Ramachandran allowed %	8.26			7.13	6.65	6.33		4.74
Ramachandran outliers %	0.87			0.7	0.28	0.66		0.51
Rotamers outliers %	0.75			0.25	0.45	0.62		0.25
MolProbity score	2.08			1.97	1.93	1.89		1.82
Clashscore	10.8			9.26	9.16	8.22		8.52
CC (mask)	0.83			0.73	0.62	0.78		0.25
CC (box)	0.39			0.54	0.4	0.5		0.34
CC (peaks)	0.43			0.39	0.28	0.46		0.18
CC (volume)	0.82			0.73	0.62	0.76		0.27
Mean CC for Ligands	0.76			0.62		0.71		0.23

Appendix Table S3: CryoEM data collection, processing and modeling statistics
