# Supplementary material for: Structural insights into the mechanism of DNA branch migration during homologous recombination in bacteria
Source: EMBO J. 2024 Oct 18;43(23):6180–98. doi: 10.1038/s44318-024-00264-5 (PMC11612176; doi:10.1038/s44318-024-00264-5)
Supplement: Supplementary file 3 — Movie EV1 [file 44318_2024_264_MOESM3_ESM.zip › Movie EV1 legend.docx]

Movie EV1**: 360-degree rotation of RadA, depicted as transparent ribbons, with only the DNA binding loops colored in the color-scheme of figure 2.** DNA backbone is shown in ribbon representation, with magenta color for 5’->3’ strand and golden for 3’->5’ strand. Nucleotide bases are depicted as blue rectangles.
