# Supplementary figures and images for: Structural insights into the mechanism of DNA branch migration during homologous recombination in bacteria
Source: EMBO J. 2024 Oct 18;43(23):6180–98. doi: 10.1038/s44318-024-00264-5 (PMC11612176; doi:10.1038/s44318-024-00264-5)

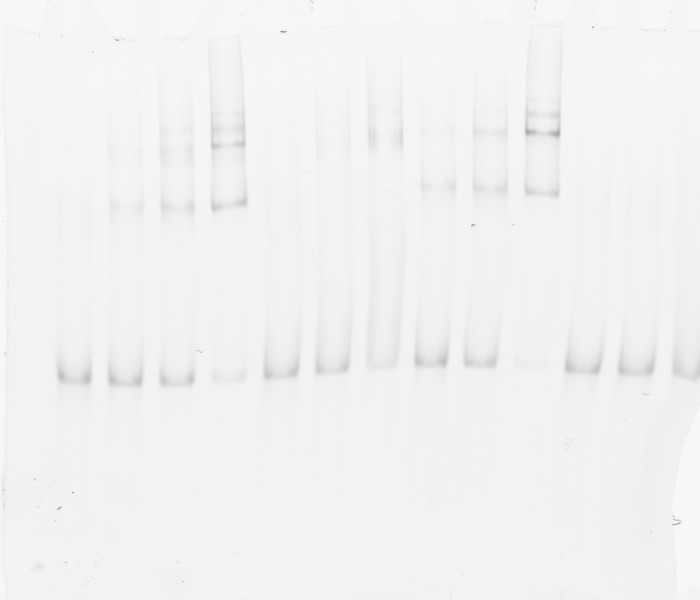

Supplement: Supplementary file 5 — Source data Fig. 5 [file 44318_2024_264_MOESM5_ESM.zip › SourceData/Figure5/5B/dsDNA-gel.tif]

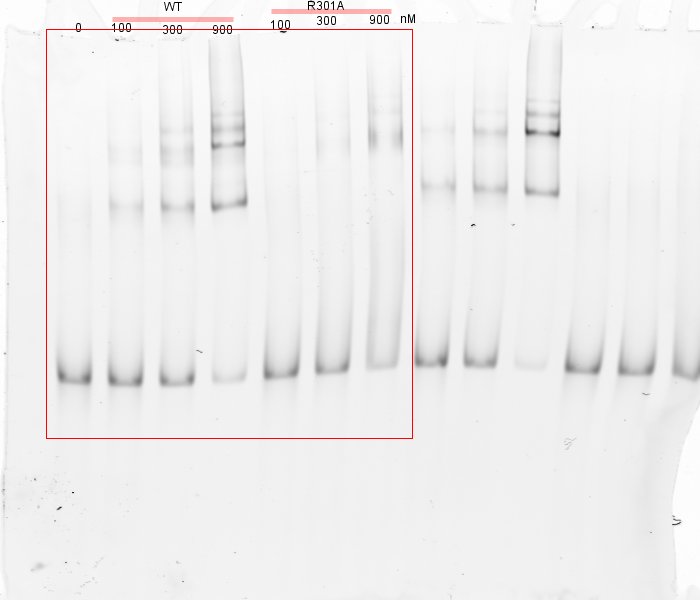

Supplement: Supplementary file 5 — Source data Fig. 5 [file 44318_2024_264_MOESM5_ESM.zip › SourceData/Figure5/5B/dsDNA-gel_annotation.jpg]

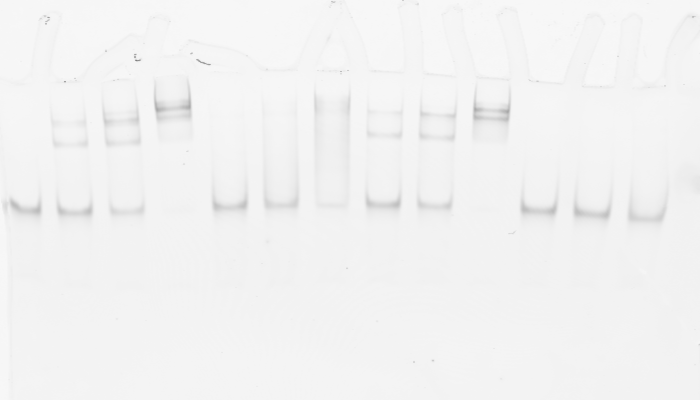

Supplement: Supplementary file 5 — Source data Fig. 5 [file 44318_2024_264_MOESM5_ESM.zip › SourceData/Figure5/5B/ssDNA-gel.tif]

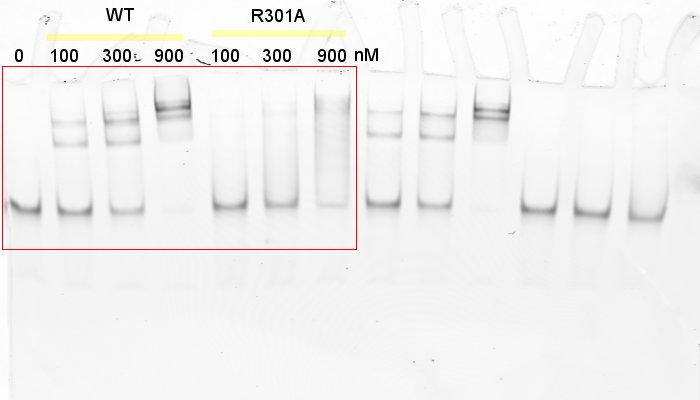

Supplement: Supplementary file 5 — Source data Fig. 5 [file 44318_2024_264_MOESM5_ESM.zip › SourceData/Figure5/5B/ssDNA-gel_annotation.jpg]
