# Supplementary material for: Structural insights into the mechanism of DNA branch migration during homologous recombination in bacteria
Source: EMBO J. 2024 Oct 18;43(23):6180–98. doi: 10.1038/s44318-024-00264-5 (PMC11612176; doi:10.1038/s44318-024-00264-5)
Supplement: Supplementary file 6 — Expanded View Figures [file 44318_2024_264_MOESM6_ESM.pdf]

Expanded View Figures

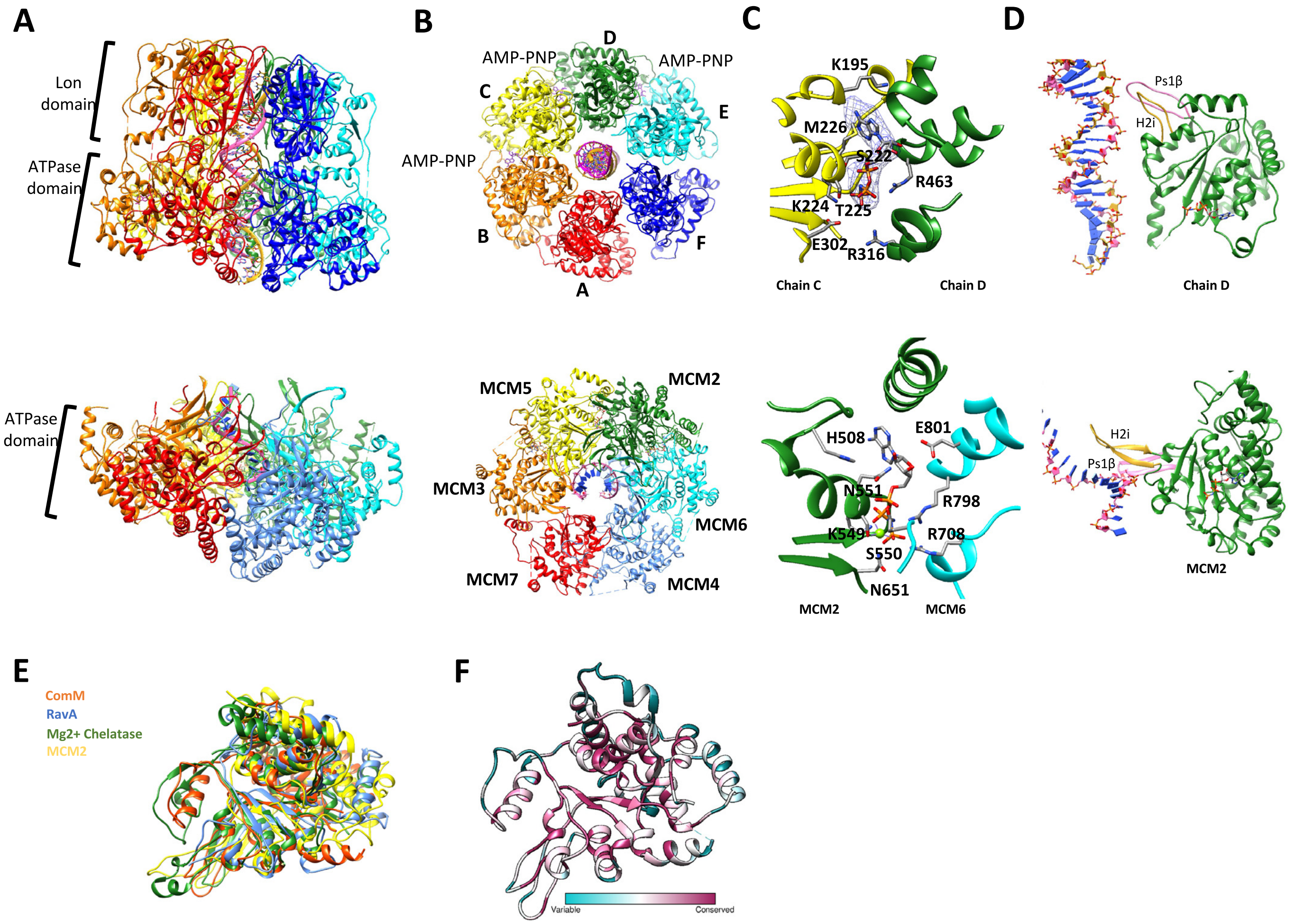

Figure EV1. Comparison between ComM and the C-terminal ATPase domains of human MCM (PDB: 6SKL), RavA (PDB 6SZB) and magnesium chelatase (PDB 1G8P).

(**A**) Arrangement around the DNA for ComM (top) and MCM (bottom), with MCM colored according to ComM scheme, from MCM7 to MCM4. (**B**) View from the top on the N-terminal side of ComM (top) and MCM (bottom). (**C**) ATP coordination site in ComM (top) and MCM (bottom). In ComM, AMPPNP is depicted in sticks, colored by element, with the cryoEM density map depicted as a blue mesh. Chains C and D are depicted as yellow and green ribbons, respectively, with relevant residues depicted in sticks and colored by element. In MCM, MCM2 is depicted in green, while MCM6 is depicted in cyan. AMPPNP is depicted as sticks, colored by element, and so are relevant residues. (**D**) Comparison between DNA-binding loops of ATPase domains in ComM (top) and MCM (bottom). The ATPase domains of ComM monomer D and MCM2 are depicted in green ribbons, with ps1B loop depicted in pink and H2i in golden. (**E**) Superposition of ComM, RavA, Mg^{2+}-chelatase and MCM2. ComM ATPase domain (residues 190–500) is shown in orange, RavA ATPase domain is depicted in blue (PDB 6SZB, residues 3–306), and shows an RMSD of 2.176 Å with ComM. Mg^{2+} Chelatase (PDB 1G8P) is shown in green, and has an RMSD of 1.928 Å with ComM. MCM2 ATPase domain is depicted in yellow (PDB 6RAW- chain 2, residues 447–798), and has a RMSD of 1.5 Å over 56 pruned atoms. (**F**) Consurf analysis of ComM ATPase domain (residues 190–500), showing conservation of residues along homologous sequences, depicted in a color gradient from cyan (variable) to purple (conserved).

 © The Author(s)

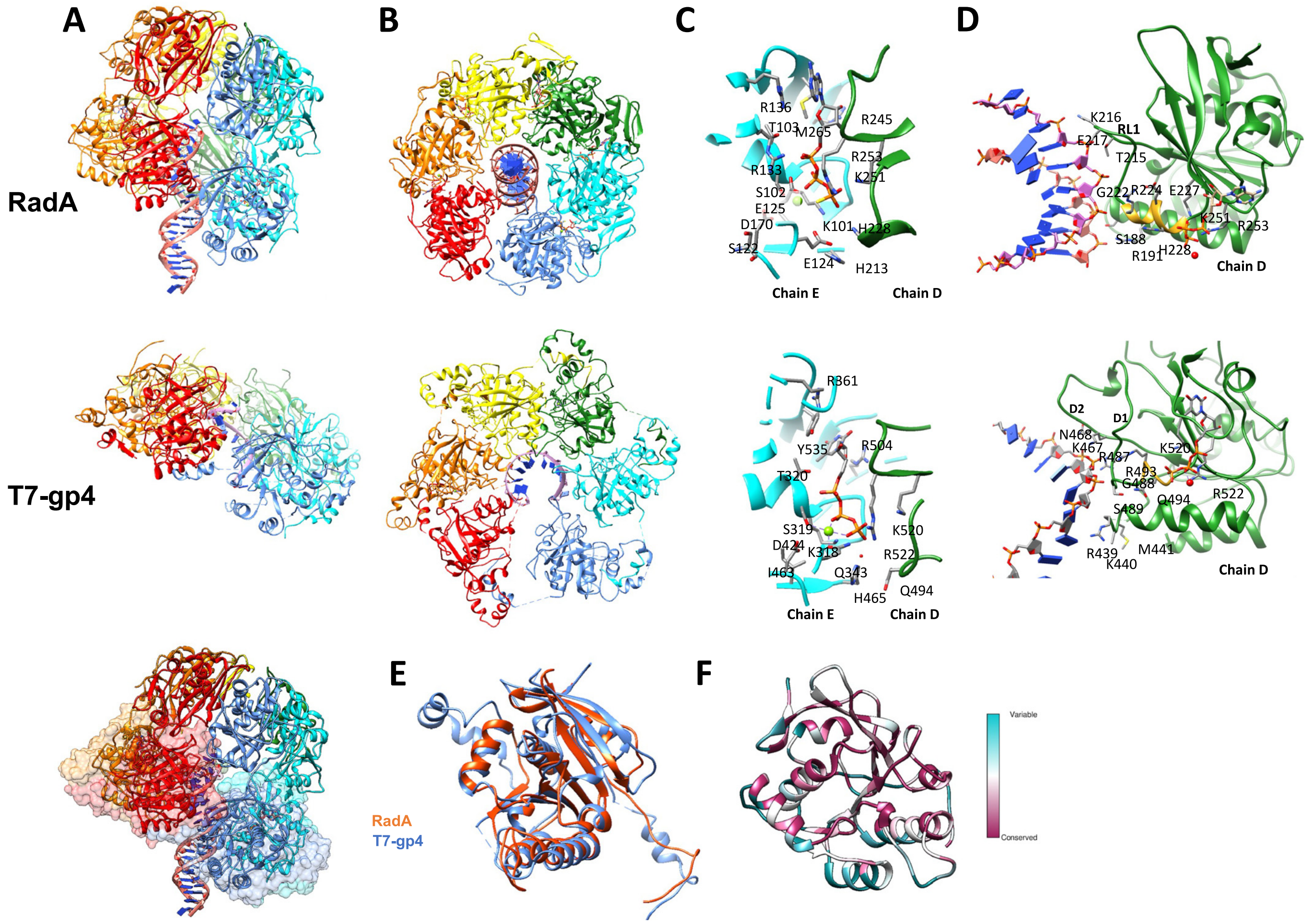

Figure EV2. Comparison between RadA and the ATPase region of the superfamily 4 helicase T4-gp4 (PDB: 6N7I).

(**A**) Overall structure of RadA (top) and T7-gp4 (middle). Both helicases present a helical arrangement, tilted in relation to the DNA helix. Bottom panel depicts RadA in ribbon representation, fit inside the transparent surface density of T7-gp4. (**B**) Top view from the C-terminaus of RadA (top panel) and T7-gp4 (bottom panel). (**C**) ATP coordination sites of RadA (top) and T7-gp4 (bottom). ATP-γ-S (in RadA) and ATP (in T7-gp4) are depicted as sticks colored by element. Ribbon representation for Chain D is shown in green and chain E in cyan. Relevant residues are shown as sticks, colored by element. (**D**) Cooperative coordination of ATP and DNA in RadA (top) and T7-gp4 (bottom). ATP-γ-S (in RadA) and ATP (in T7-gp4), as well as relevant residues, are depicted in stick representation colored by elements. The ATPase domains are depicted as green ribbon, with the region structured upon ATP binding highlighted in golden. DNA is depicted as sticks, with the bases represented as blue rectangles. (**E**) Superposition of the ATPase domains of RadA (orange, residues 54–273) and T7-gp4 (blue, residues 263–547), resulting in an RMSD of 1.16 Å over 120 pruned residues or 1.944 Å overall. (**F**) ConSurf analysis of the ATPase domain of RadA, showing conservation of residues along homologous sequences, depicted in a color gradient from cyan (variable) to purple (conserved).

© The Author(s)
